# Does Multiparametric Magnetic Resonance of Prostate Outperform Risk Calculators in Predicting Prostate Cancer in Biopsy Naïve Patients?

**DOI:** 10.3389/fonc.2020.603384

**Published:** 2021-01-08

**Authors:** Ugo Giovanni Falagario, Giovanni Silecchia, Salvatore Mariano Bruno, Michele Di Nauta, Mario Auciello, Francesca Sanguedolce, Paola Milillo, Luca Macarini, Oscar Selvaggio, Giuseppe Carrieri, Luigi Cormio

**Affiliations:** ^1^ Department of Urology and Organ Transplantation, University of Foggia, Foggia, Italy; ^2^ Department of Urology, Bonomo Teaching Hospital, Andria, Italy; ^3^ Department of Pathology, University of Foggia, Foggia, Italy; ^4^ Department of Radiology, University of Foggia, Foggia, Italy

**Keywords:** prostate cancer, mpMRI, decision curve analysis, clinically significant prostate cancer, risk calculator

## Abstract

**Background:**

European Association of Urology (EAU) guidelines recommend using risk-calculators (RCs), imaging or additional biomarkers in asymptomatic men at risk of prostate cancer (PCa).

**Objectives:**

To compare the performance of mpMRI, a RC we recently developed and two commonly used RC not including mpMRI in predicting the risk of PCa, as well as the added value of mpMRI to each RC.

**Design, Setting, and Participants:**

Single-center retrospective study evaluating 221 biopsy-naïve patients who underwent prebiopsy mpMRI.

**Outcome Measurements and Statistical Analysis:**

Patients’ probabilities of any PCa and clinically significant PCa (csPC, defined as Gleason-Score ≥3 + 4) were computed according to mpMRI, European Randomized Study of Screening for Prostate Cancer RC (ERSPC-RC), the Prostate Biopsy Collaborative Group RC (PBCG-RC) and the Foggia Prostate Cancer RC (FPC-RC). Logistic regression, AUC, and Decision curve analysis (DCA) were used to assess the accuracy of tested models.

**Results and Limitation:**

The FPC-RC outperformed mpMRI in diagnosing both any PCa (AUC 0.76 *vs* 0.69) and csPCa (AUC 0.80 *vs* 0.75). Conversely mpMRI showed a higher accuracy in predicting any PCa compared to the PBCG-RC and the ERSPC-RC but similar performances in predicting csPCa. At multivariable analysis predicting csPCa and any PCa, the addition of mpMRI findings improved the accuracy of each calculator. DCA showed that the FPC-RC provided a greater net benefit than mpMRI and the other RCs. The addition of mpMRI findings improved the net benefit provided by each calculator.

**Conclusions:**

mpMRI was outperformed by the novel FPC-RC and showed similar performances compared to the PBCG and ERSPC RCs in predicting csPCa. The addition of mpMRI findings improved the diagnostic accuracy of each of these calculators

## Introduction

In current clinical practice, the cancer detection rate (CDR) of a first extended prostate biopsy (PBx) prompted by an elevated serum prostate-specific antigen (PSA) level and/or an abnormal digital rectal examination (DRE) is around 40%, dropping to approximately 25% in the setting of screening programs, *i.e.* patients with serum PSA between 2.5 and 10 ng/ml (1, 2).

To reduce the risk of unnecessary PBxs, current European Association of Urology (EAU) guidelines (3) provide a strong recommendation to offer further risk-assessment to asymptomatic men with normal DRE but PSA levels between 2 and 10 ng/ml prior to performing PBx. Such “further risk assessment” should be done by one of following tools: i) risk-calculator (RC); ii) imaging; iii) an additional serum or urine-based test (3). Interestingly, while this recommendation has remained unchanged in 2018 and 2019 Guidelines, the 2020 Guidelines provide a weak recommendation to perform mpMRI in any patient with clinical suspicion for prostate cancer (PCa). If mpMRI demonstrates lesion(s) suspicious for PCa, systematic and target biopsy should be performed, whereas biopsy can be avoided when mpMRI is negative and the clinical suspicion of PCa is low. By doing so, the 2020 Guidelines somehow bind the decision to perform mpMRI to the clinical suspicion of PCa which is well determined by available RCs.

RCs are designed to determine the risk of an individual harboring PCa by entering into a statistical model his clinical parameters. To date, several calculators have been developed and externally validated; a few also include mpMRI findings and biomarkers but questions remain on the additional value provided by such tests. Indeed, a recent study aiming at comparing and externally validating prostate cancer RCs incorporating mpMRI demonstrated that the addition of mpMRI parameters to RCs based on standard clinical variables was limited (3). Overall, available information regarding the use of RCs, mpMRI or biomarkers as triage test and the utility of combining them remain scarce.

The present study therefore aimed to compare the performance of mpMRI with the performances of two commonly-used externally-validated calculators not including mpMRI (4, 5) and a novel externally-validated calculator we recently developed (6) in predicting the risk of harboring PCa, as well as the added value of mpMRI to each RC.

## Patients and Methods

Our Internal Review Board which approved the database on prostate biopsy was queried to identify patients who underwent mpMRI and trans-rectal prostate biopsy at our institution under the clinical suspicion of PCa. The patient population used for the development of our RC was not included in the present study.

Prostate mpMRI was triggered by PSA higher than 3.0 ng/ml and/or abnormal DRE and were interpreted by a single dedicated radiologist (PM) with 10 years of experience in prostate MRI, using the PIRADSv2.0 recommendations (7).

All patients underwent PSA measurement before DRE and transrectal ultrasound (TRUS). Uroflowmetry (UFM) was carried out before PBx, waiting for the patient to report a strong sensation to void.

MRI examinations were performed using a 1.5 T MR scanner (Achieva, Philips Healthcare, Best, The Netherlands) and surface array coils (SENSE Flex surface) or with endorectal coil (ERC) combined with 16-channel surface coil (TORSO-XL coil). The mpMRI protocol was compliant with PIRADs 2.0 recommendations (7) and consisted of: A. Turbo-Spin-Echo (TSE) T2-weighed imaging in axial, coronal and sagittal planes [repetition time (TR) 5,300, echo time (TE) 150 ms, slice thickness 3 mm, field of view (FOV) 180 × 180, number of signal averaged (NSA) 8]; B. TSE T1-weighed imaging in axial plane [TR/TE 400–650/12 ms, thickness 3 mm, FOV 180 × 180, NSA 3]; C. Diffusion-weighted imaging sequence (DWI) in the axial plane [TR/TE 3,481/92 ms, slice thickness 3 mm, FOV 180 × 220, NSA 4, b-values 0–500–1000–1,500/2,000 s/mm^2^]; D. Dynamic contrast enhanced prostate MRI was performed using a T1-weighted high resolution isotropic volume examination (THRIVE) on the axial plane [TR/TE 4.5/2.2 ms, slice thickness 3 mm, FOV 184 × 220, NSA 1] following injection of 0.1 ml/kg of gadobutrol followed by 20 ml of saline solution using an automatic injector at a rate of 2 ml/s.

In accordance with the current EAU guidelines, patients with negative mpMRI (PIRADS 1 and 2 lesions were considered to be negative**)** received a standard ultrasound guided transrectal PBx using our 18-core template (8); those with a positive mpMRI received a transrectal electromagnetic-tracked MRI/US fusion guided biopsy (Navigo, UC-CARE, Yokneam, ISR). To avoid large differences in the number of cores, we attempted to include the two target cores from each mpMRI-suspicious lesions into our 18-core biopsy scheme (SBx). All procedures were carried out by two of us (OS, GS) under local non-infiltrative anesthesia (8, 9).

A single, dedicated uropathologist (FS) reviewed all biopsy specimens according to International Society of Urological Pathology; Gleason Grade Groups (GGG) were assigned to each patient (10). Contemporary diagnostic criteria for high-grade prostatic intraepithelial neoplasia (HGPIN), atypical small acinar proliferation (ASAP) of prostate (11), and PCa were followed.

### Statistical Analysis

Outcomes of the present study were probabilities of any PCa (GGG≥1) and clinically significant PCa (csPC defined as GGG≥2) as assessed by mpMRI alone, or by one RC alone, namely the European Randomized Study of Screening for Prostate Cancer (ERSPC) (4), the Prostate Biopsy Collaborative Group (PBCG) (5) and the Foggia Prostate Cancer (FPC) RCs (6), or by adding mpMRI to each RC.

Our primary objective was to compare the accuracy of mpMRI and the FPC-RC. As a secondary objective we sought to compare the accuracy of mpMRI with two of the most used available RCs. Finally, we determined the added value of mpMRI to each of the tested models.

For descriptive statistics, continuous variables were reported as medians and interquartile ranges, whereas categorical variables were reported as rates. Patients’ probabilities of any PCa as well as csPCa were computed applying the coefficients **(**available upon request to the authors of the original publications) to the logit functions for the ERSPC-RC and the FPC-RC. Conversely, individual probability of i. No cancer, ii. Low grade cancer, and iii. High grade cancer for the PBCG-RC was computed using the coefficients and formulas provided by the authors as supplementary materials (5).

Univariable logistic regression was carried out in order to compare each RC against mpMRI as predictors of the outcomes of interest.

Three models predicting any PCa and three models predicting csPCa were created adding mpMRI to the individual risk computed for each calculator, in a multivariable model. Since the PBCG-RC was developed using multinomial regression (*i.e.* it provides risk of no cancer, low-grade cancer, high-grade cancer), the risk prediction for any cancer was computed as 1-risk of no cancer and was identical to the risk of low-grade cancer + risk of high-grade cancer.

The corresponding area under receiver operating characteristic (ROC) curve (AUC) and decision curve analysis (DCA) were used to assess the predictive accuracy and clinical benefit of tested models.

Statistical analyses were performed according to the latest guidelines (12) using STATA 15 (StataCorp LP, College Station, TX, USA). Significance was set at *α* = 0.05.

## Results

Between January 2017 and October 2019, a total of 415 patients underwent mpMRI and PBx at our Institution. Men receiving five alfa-reductase inhibitors (N = 50), or who had previously undergone PBx (N = 174), or invasive treatment for benign prostatic hyperplasia (n = 11), or with dwelling urethral catheters (N = 5), or with a voided volume of less than 150 ml (N = 7) were excluded from the present study. Patients with PSA >20 ng/ml (N = 18) were also excluded as we found them to have a too high risk (>75%) of harboring PCa.

After the exclusion criteria, the final population included 221 biopsy naïve patients with complete data. Patients characteristics are summarized in **Table 1**; 43 patients (19.5%) had a negative mpMRI (PIRADS 1–2), thus underwent 18-core US guided transrectal PBx, whereas the remaining 168 underwent mpMRI/US guided fusion PBx. Their PIRADS score was 3, 4, and 5 in 35 (15.8%), 120 (54.3%) and 23 (10.4%), respectively.

**Table 1:** Descriptive characteristics of the study population.

	Overall populationN = 221
**Age**	66.0 (60.0, 71.0)
**DRE, n (%)**	
Negative	123 (55.7%)
Suspicious	98 (44.3%)
**Family History**	
Negative	108 (76.1%)
Positive	34 (23.9%)
**PSA, ng/ml**	5.5 (4.1, 7.4)
**PSA density**	0.11 (0.07, 0.15)
**Prostate volume, cc**	52.0 (39.0, 69.0)
**PIRADS**	
1-2	43 (19.5%)
3	35 (15.8%)
4	120 (54.3%)
5	23 (10.4%)
**Any Cancer**	119 (53.8%)
**Cs Cancer**	60 (27.1%)
**NPV of PIRADS 1-2**	97.8% (42/43)
**PPV of PIRADS 3-4-5**	33.1% (59/178)

Any PCa and csPCa detection rates were 53.8% (n = 119) and 27.1% (n = 60), respectively. The negative predictive value of mpMRI (PIRADS 1–2) in predicting any PCa and csPCa was 76.7 and 97.7% respectively. The positive predictive value of mpMRI (PIRADS 3–4–5) was 61.2% for any PCa and 33.1% for csPCa.

Univariable analysis predicting the outcomes of interest is shown in **Table 2**. RCs and mpMRI PIRADS score were all significant predictors of any PCa and csPCa (p < 0.05).

**Table 2:** Univariable analysis predicting any cancer and csPCa using risk calculators and mpMRI.

	OUTCOME: ANY PCa				OUTCOME: csPCa			
	O.R.	95% CI	P>|z|	AUC	O.R.	95% CI	P>|z|	AUC
**FPC-RC, per unit**	1.05	1.03,1.07	<0.001	0.760	1.06	1.04,1.08	<0.001	0.801
**PBCG-RC, per unit**	1.03	1.01,1.05	0.001	0.615	1.05	1.03,1.07	<0.001	0.733
**ERSPC-RC, per unit**	1.00	1.00,1.00	0.001	0.614	1.09	1.06,1.13	<0.001	0.749
**MRI highest PIRADS**								
1-2	Ref.			0.690	Ref.			0.754
3	2.20	0.83,5.85	0.114		1.24	0.07,20.49	0.883	
4	5.91	2.66,13.15	<0.001		24.32	3.23,182.86	0.002	
5	11.88	3.52,40.14	<0.001		65.33	7.59,562.40	<0.001	

**Figure 1:**
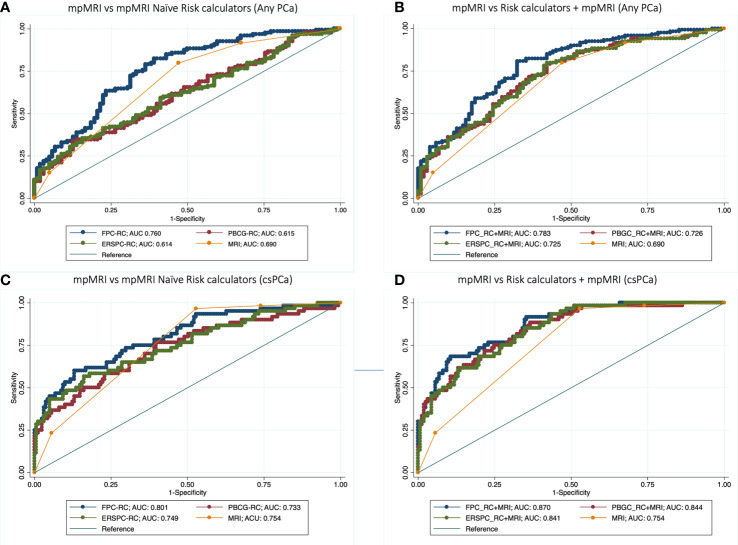
Receiver operator curve (ROC) analysis comparing accuracy of mpMRI *vs* mpMRI naïve Risk calculators (RCs) **(A–C)**, and model based on mpMRI + RCs **(B–D)** for detecting any PCa **(A, B)** and clinically significant prostate cancer **(C, D)**.

The FPC-RC outperformed mpMRI in diagnosing both any PCa (AUC 0.76 *vs* 0.69) and csPCa (AUC 0.80 *vs* 0.75). Conversely mpMRI showed a higher accuracy in predicting any PCa compared to the PBCG-RC and the ERSPC-RC but similar performances in predicting csPCa (**Table 2**, **Figures 1A–C**).

Multivariable analysis showed that the addition of mpMRI findings improved the diagnostic accuracy of each calculator in predicting both csPCa and any PCa. The model derived from the addition of mpMRI to the FPC-Rc showed the highest accuracy in diagnosing both any PCa (AUC 0.78) and csPCa (AUC 0.87) (**Table 3**, **Figures 1B, D** **)**.

**Table 3:** Multivariable analysis predicting any PCa and csPCa.

	OUTCOME: ANY PCa				OUTCOME: csPCa			
	O.R.	95% CI	P>|z|	AUC	O.R.	95% CI	P>|z|	AUC
**MODEL-1**								
**FPC-RC, per unit**	1.04	1.03,1.06	<0.001	0.783	1.06	1.04,1.08	<0.001	0.870
**MRI highest Pirads**								
1-2	Ref.				Ref.			
3	1.91	0.67,5.47	0.226		1.67	0.08,33.59	0.738	
4	3.51	1.47,8.40	0.005		25.74	2.67,247.97	0.005	
5	6.42	1.73,23.89	0.006		60.17	5.18,698.68	0.001	
**MODEL-2**								
**PBCG-RC, per unit**	1.03	1.00,1.05	0.015	0.726	1.05	1.03,1.07	<0.001	0.844
**MRI highest Pirads**								
1-2	Ref.				Ref.			
3	2.28	0.85,6.14	0.104		1.30	0.08,22.33	0.855	
4	5.59	2.49,12.55	<0.001		23.75	3.06,184.34	0.002	
5	9.78	2.84,33.63	<0.001		51.87	5.73,469.84	<0.001	
**MODEL-3**								
**ERSPC-RC, per unit**	1.00	1.00,1.00	0.012	0.725	1.08	1.04,1.12	<0.001	0.841
**MRI highest Pirads**								
1-2	Ref.				Ref.			
3	2.29	0.85,6.17	0.102		1.66	0.09,30.08	0.731	
4	5.58	2.48,12.54	<0.001		24.23	2.87,204.38	0.003	
5	9.66	2.80,33.28	<0.001		45.37	4.62,445.97	0.001	

Three models were created adding mpMRI to each risk calculator.

**Figure 2:**
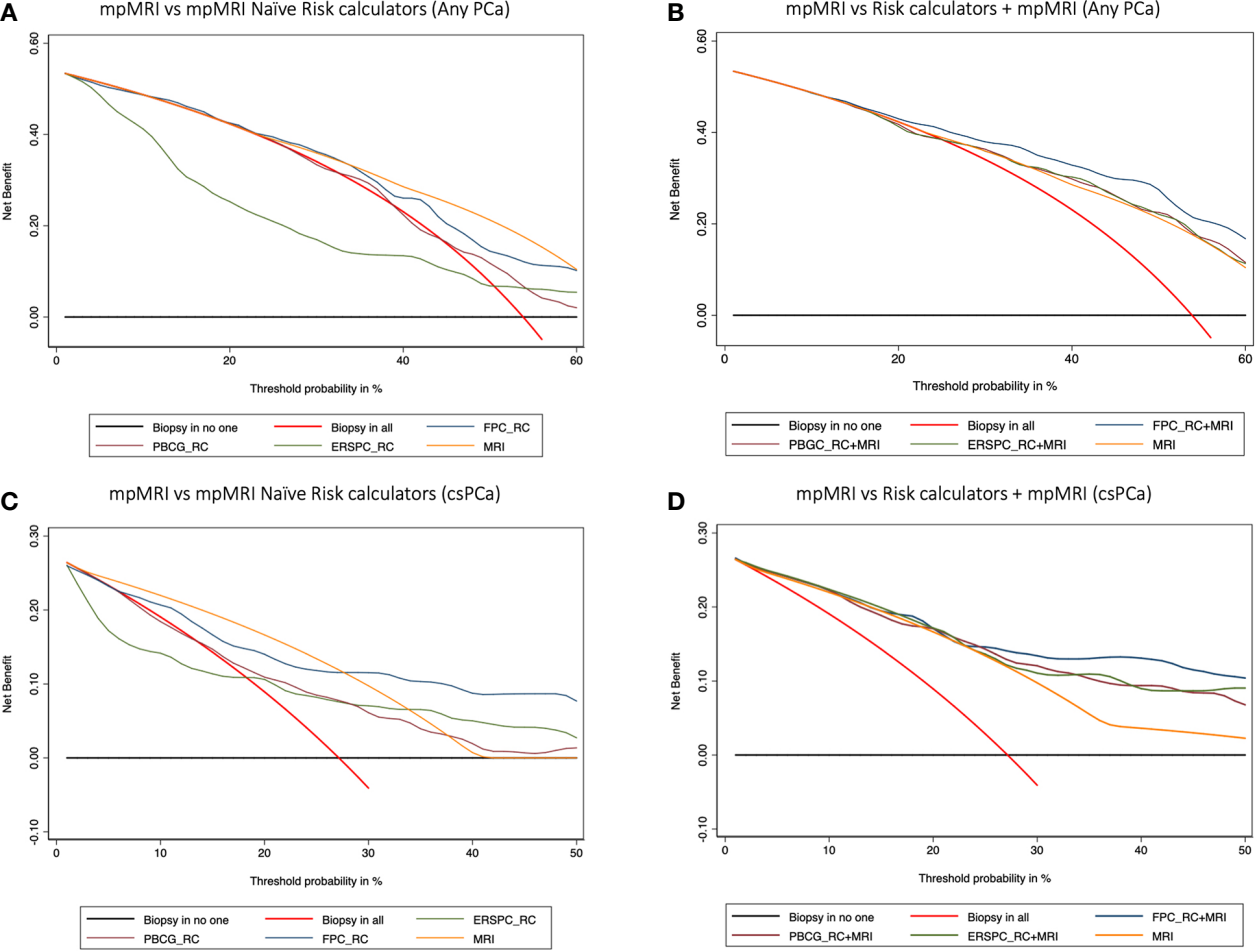
Decision curve analysis (DCA) comparing the clinical utility of mpMRI *vs* mpMRI naïve risk calculators (RCs) **(A–C)**, and model-based on mpMRI + RCs **(B–D)** for detecting any PCa **(A, B)** and clinically significant prostate cancer **(C, D)**. The DCA simulates two scenarios: one in which all patients would receive biopsy (biopsy in all) and one in which none undergoes biopsy (biopsy in no one). Clinically useful models lie above these scenarios. Models including mpMRI +RCs showed a higher net benefit at each threshold probability and thus outperformed mpMRI alone in determining the need for a prostate biopsy.

Finally, DCA showed that the FPC-RC provided greater net benefit than mpMRI in predicting any PCa and csPCa. Conversely mpMRI had a higher net benefit compared to the other RCs. Again, the addition of mpMRI findings improved the net benefit provided by each calculator benefit (**Figure 2**).

## Discussion

Over the last years, mpMRI has gained popularity as a reliable tool in localizing specific regions of the prostate highly suspicious for csPCa; therefore, there is a trend to recommend it as the most efficient tool in predicting PCa at PBx (13). Such recommendation is however based on prospective studies in high volume tertiary cancer centers that do not reflect everyday practice in less experienced centers (14–16). Indeed, mpMRI suffers a great inter-reader and inter-center variability (13, 17, 18); moreover, it is expensive. and not all institutions may afford to test every patient at risk for PCa. Conversely, RCs are freely available online and have been proved to be effective in several external validation cohorts.

The first interesting finding of our study was that the FPC-RC, which has recently been externally validated in a cohort of 1,377 biopsy naïve patients from 11 institutions (19), outperformed mpMRI in predicting PBx outcomes. This finding somehow further supports the clinical value of benign prostatic obstruction parameters in the evaluation of patients with PCa suspicion (20–22). Differently from mpMRI, the FPC-RC is a freely available and almost inexpensive tool that can be easily used during any medical consultation. Should our findings be replicated in further external cohorts, the FPC-RC may become an essential tool for patients requiring “further risk assessment” prior to performing PBx.

When compared to other RCs, mpMRI outperformed the ERSPC-RC and the PBCG-RC in predicting any PCa but showed similar performances in predicting csPCa.

Our study also aimed to answer the relevant question whether combining diagnostic tools may improve their diagnostic accuracy. Overall, the addition of mpMRI findings improved the diagnostic accuracy of each calculator in predicting both csPCa and any PCa. Our findings are in line with those from a single center study whereby the diagnostic accuracy of 4 RCs incorporating mpMRI (23–26) was compared with that of the ERSPC-RC (4) and PBCG-RC (5) in a population of 468 patients. The four RCs incorporating mpMRI parameters provided better discrimination, calibration, and clinical usefulness; however, none of the six calculators demonstrated clinical benefit against a “biopsy all” strategy at thresholds of less than 15% (27). This finding underlines a potentially relevant limitation of RCs; specifically, a model that shows benefit at high thresholds of probability is clinically useless in a screening setting since the decision to perform a biopsy is especially difficult in patients with borderline risk.

In the present study, DCA showed that the combination of mpMRI and RCs provided a greater benefit than the “biopsy all” strategy at low thresholds. Having said this, additional external validation studies in different biopsy settings are warranted since the clinical utility of these models could be cohort dependent. It is also worth mentioning that calculators including mpMRI, though outperforming the mpMRI naïve ones, involve obtaining mpMRI in all patients and this may not be afforded in centers with limited resources (28). Conversely, mpMRI naïve RCs offer the unique opportunity to potentially tailor further testing, such as mpMRI and PBx itself, on an individual basis. Indeed, it has been pointed out that RCs and biomarkers may help in selecting patients who could benefit from mpMRI and PBx and patients with a very low risk of csPCa in whom the positive predictive value of mpMRI is low and mpMRI and PBx should be avoided (29–31).

The findings of this study have to be seen in light of some limitations. First, the FPC-RC was developed at our institution, and this can explain its better performance compared to the other tested RCs. Even if the patient population used for the development of the RC was not included in the present study, this cannot be considered an external validation study. Other potential study limitations include its relatively small sample size and its retrospective nature; however, we elected to use strict inclusion criteria and data were prospectively collected. Finally, we did not test novel and promising tools such as bi-parametric MRI (32) and novel biomarkers (33), but this would have been beyond the aim of a study comparing currently available tests.

## Conclusions

The present study pointed out that mpMRI was outperformed by the novel FPC-RC and showed similar performances compared to the PBCG and ERSPC risk calculators in predicting csPCa. The addition of mpMRI findings improved the diagnostic accuracy of each of these calculators. Further studies are needed to assess how these findings can be used to safely avoid unnecessary biopsies.

## Data Availability Statement

The raw data supporting the conclusions of this article will be made available by the authors, without undue reservation.

## Ethics Statement

The studies involving human participants were reviewed and approved by the University of Foggia Ethics committee. The patients/participants provided their written informed consent to participate in this study.

## Author Contributions

LC, UF, GC, LM, and OS conceived and designed the study. GS, MB, MN, MA, and PM acquired the data. LC, UF, and PM analyzed and interpreted the data. LC, UF, GS, and MB drafted the manuscript. LC, GC, OS, and LM made critical revisions of the manuscript for important intellectual content. LC and UF conducted the statistical analysis. LC, GC, OS, and LM supervised the study. All authors contributed to the article and approved the submitted version.

## Conflict of Interest

The authors declare that the research was conducted in the absence of any commercial or financial relationships that could be construed as a potential conflict of interest.
